# Novel role of circRNAs in the drug resistance of gastric cancer: regulatory mechanisms and future for cancer therapy

**DOI:** 10.3389/fphar.2024.1435264

**Published:** 2024-09-09

**Authors:** Ling Lu, Zihan Gao, Longtao Jin, Hao Geng, Zhaofeng Liang

**Affiliations:** ^1^ Child Healthcare Department, The Fourth Affiliated Hospital of Jiangsu University, Zhenjiang, Jiangsu, China; ^2^ Jiangsu Key Laboratory of Medical Science and Laboratory Medicine, School of Medicine, Jiangsu University, Zhenjiang, Jiangsu, China; ^3^ Department of Urology, The Second Affiliated Hospital of Anhui Medical University, Hefei, Anhui, China

**Keywords:** gastric cancer, circRNAs, drug sensitivity, cancer therapy, mechanism

## Abstract

Cancer, including gastric cancer, has become a serious disease that jeopardizes public life. Currently, the main treatment methods are surgery, radiation therapy, and chemotherapy. One of the primary causes of death for patients with gastric cancer is drug resistance. Several mechanisms of anticancer drugs resistance have been reported, including changes in drugs transport and metabolism, mutations in drug targets, changes in DNA repair systems, inhibition of cell apoptosis and autophagy, gastric cancer stem cells, invasion and migration. It is becoming more widely known that non-coding RNAs, like circRNAs, play a critical role in the resistance of drugs used to treat gastric cancer. CircRNAs have a unique structure and function that is related to gastric cancer resistance, cell proliferation, apoptosis, autophagy, DNA repair systems, migration, and invasion. A clear understanding of the molecular mechanism of circRNAs mediated the resistance of gastric cancer drugs will open a new window for the treatment and management of gastric cancer. Therefore, in this review, we will summarize the current mechanism of drug resistance, and finally discuss the molecular mechanism of circRNAs in regulating the development of drug resistance in gastric cancer.

## 1 Introduction

Gastric is a common malignant tumor of the digestive tract that originates from the gastric mucosa, which is extremely dangerous to people’s health. According to statistics, there are approximately 1.089 million new cases and 768,000 deaths worldwide each year (Siegel et al., 2023; Sung et al., 2021).

The majority of gastric cancer patients are discovered in the late stage and have a bad prognosis because there aren’t many clear-cut, particular stage symptoms in the early stages of the disease. For patients with advanced gastric cancer, the use of combined chemotherapy and targeted therapy dramatically improves overall survival and quality of life (Wei et al., 2020). However, the use of anti-cancer drugs reduces the sensitivity of gastric cancer cells to drugs, gradually leading to drug resistance in cancer cells. The decrease in drug sensitivity and drug resistance is a major obstacle in clinical oncology, which can result in a poor prognosis for patients with gastric cancer. The complex mechanisms involved in drug resistance in gastric cancer involve various pathological and physiological processes, including cell apoptosis, cell cycle, tumor heterogeneity, cell proliferation, autophagy, DNA damage repair, tumor microenvironment regulation of drug intake and/or drug efflux related proteins, gastric cancer stem cells, and EMT (Wei et al., 2020; Wang et al., 2022a; Zheng et al., 2023). However, the detailed mechanism of drug resistance remains inconclusive. Studies have shown that non coding RNAs such as circRNAs play an important regulatory role in drug resistance and sensitivity in gastric cancer cells.

**FIGURE 1:**
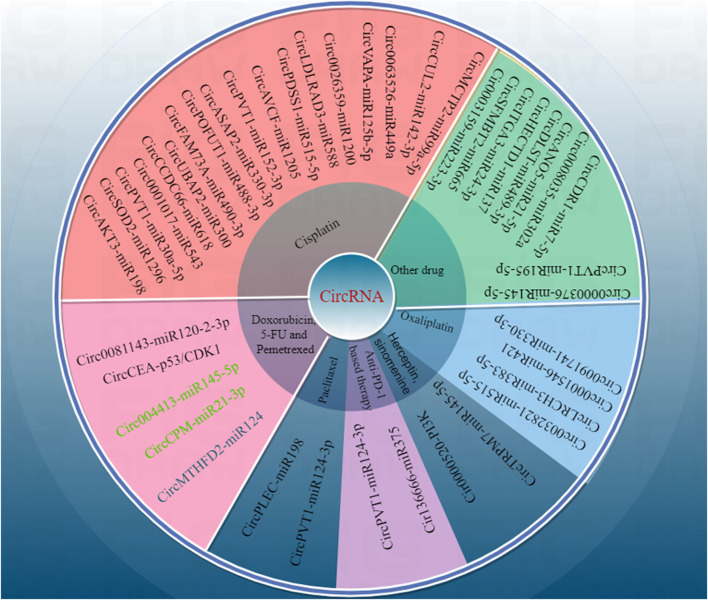
By Figdraw. CircRNA affected the sensitivity and mechanism of gastric cancer to various anti-cancer drugs.

Circular RNAs, as a new class of ncRNAs, are more stable than linear RNAs due to their unique structure (Samavarchi Tehrani et al., 2023; Wang et al., 2022b; Mu et al., 2021). More and more evidence suggests that circRNA can play a crucial role in various cancer biology, such as gastric cancer, by regulating the biological characteristics of gastric cancer cells such as sensitivity, proliferation, migration, and invasion to anticancer drugs through different molecular mechanisms (Samavarchi Tehrani et al., 2023; Wang et al., 2022b; Ma et al., 2024; Cao et al., 2022) (Figure 1; Table 1). Therefore, a deeper understanding of the correlation between the functions of circRNAs and drug resistance in gastric cancer will open up a new development path and innovative treatment plans to overcome drug resistance in gastric cancer and enhance the drug sensitivity of gastric cancer cells. Therefore, in this review, we will summarize and discuss the role and molecular mechanisms of circRNAs in regulating drug resistance in gastric cancer cells, and explore new strategies for improving gastric cancer treatment based on research results.

**TABLE 1:** Overview of the mechanism of drug resistance in gastric cancer.

Physiological and pathological processes	CircRNA	Target	Drug	References
Migration, invasion, and autophagy	Circ0063526	MiR-449a/SHMT2	Cisplatin	Yang et al. (2022)
Invasion, proliferation, and apoptosis	CircLRCH3	MiR-383–5p/FGF7	Oxaliplatin	Xiang et al. (2023)
Migration, invasion, and proliferation	CircVAPA	MiR-125b-5p/STAT3	Cisplatin	Deng et al. (2021)
Migration and invasion	Circ0026359	MiR-1200/POLD4	Cisplatin	Zhang et al. (2020a)
Migration, invasion, and proliferation	Circ0081143	MiR-129-2-3p/YES1	Doxorubicin	Ou et al. (2022)
Migration, invasion, and proliferation	Circ0032821	MiR-515–5p/SOX9	Oxaliplatin	Zhong et al. (2021)
Invasion and proliferation	CircLDLRAD3	MiR-588/SOX5	Cisplatin	Liang et al. (2023)
Migration and invasion	CircPLEC	MiR-198	Paclitaxel	Zhou et al. (2021)
Migration, invasion, proliferation and apoptosis	CircPDSS1	MiR-515–5p and ITGA11	Cisplatin	Wang et al. (2023)
Proliferation, migration, invasion, and immune evasion	CircDLG1	CXCL12 and miR-141–3p	Anti-PD-1-based therapy	Chen et al. (2021)
Proliferation	Circ004413	MiR-145–5p	5-Fu	Zhou et al. (2022)
Proliferation and invasion	Circ-NOTCH1	FOXM1	Adriamycin	Ge et al. (2023)
Proliferation and metastasis	CircAVCF	MiR-1205/FGFR1	Cisplatin	Zhang et al. (2021)
Proliferation	Circ0012774	MiR-637/CDX2	Cisplatin	Xu et al. (2022)
Proliferation, and induces apoptosis	CircPVT1	MiR-152–3p	Cisplatin	Wang et al. (2021)
Proliferation	CircMTHFD2	MiR-124	Pemetrexed	Xu et al. (2019)
Proliferation and apoptosis	CircMCTP2	MiR-99a-5p/MTMR3	Cisplatin	Sun et al. (2020)
Proliferation, migration, invasion, and apoptosis	CircASAP2	MiR-330–3p/NT5E	Cisplatin	Sun et al. (2021)
Proliferation	Circ0001546	MiRNA-421/ATM/Chk2/p53	Oxaliplatin	Wu et al. (2020)
Autophagy	CircCUL2	MiR-142–3p/ROCK2	Cisplatin	Peng et al. (2020)
Autophagy	CircCPM	MiR-21–3p**/**PRKAA2	5-FU	Fang et al. (2022)
Autophagy	Circ0091741	MiR-330–3p/TRIM14/Dvl2/Wnt/β-catenin	Oxaliplatin	Chen et al. (2023)
Autophagy	CircPOFUT1	MiR-488–3p/PLAG1/ATG12	Cisplatin	Luo et al. (2023)
Apoptosis	CircUBAP2	MiR-300/KAT6B	Cisplatin	Cheng et al. (2023)
Apoptosis	CircCCDC66	MiR-618/BCL2	Cisplatin	Zhang et al. (2020b)
Apoptosis	Cir0000520	PI3K/Akt	Herceptin	Lv et al. (2020)
Apoptosis	CircCEA	p53/CDK1	Doxorubicin	Yuan et al. (2022)
Autophagy, invasion and apoptosis	CircPVT1	MiR-30a-5p/YAP1	Cisplatin	Yao et al. (2021)
Apoptosis and invasion	CircPVT1	MiR-124–3p/ZEB1	Paclitaxel	Liu et al. (2019)
Apoptosis and migration	Circ0001017	MiR-543/PHLPP2	Cisplatin	Zhang et al. (2022a)
DNA damage repair	CircAKT3	MiR-198/PIK3R1	Cisplatin	Huang et al. (2019)
Cancer stem cells	CircFAM73A	miR-490–3p/HMGA2 and HNRNPK/β-catenin	Cisplatin	Xia et al. (2021)
Cancer stem cells, proliferation and metastasis	Circ50547	miR-217/HNF1B		Zang et al. (2024)
Macrophage polarization	CircSOD2	miR-1296/STAT1	Cisplatin	Qu et al. (2023)
Immune escape	cir136666	miR-375/PRKDC and PD-L1	anti-PD-L1 therapy	Miao et al. (2023)
Proliferation and metastasis	CircTRPM7	miR-145–5p/PBX3	sinomenine	Yan et al. (2023)
Proliferation	Cir0003159	miR-223–3p/NLRP3	Icariin	Zhang et al. (2022b)
Proliferation, apoptosis, migration and invasion	CircSFMBT2	miR-665	paeonol	Li et al. (2020)
Proliferation and apoptosis	CircCDR1	miR-7-5p/REGγ	Diosbulbin-B	Li et al. (2019)
Proliferation	CircHECTD1	miR-137/PBX3	Diosbulbin-B	Lu et al. (2021)
Proliferation and apoptosis	CircDLST	miR-489–3p/EIF4A1	Astragaloside IV	Li et al. (2022)
Invasion and migration	CircITGA3	miR-24–3p/PDGFRB	matrine	Gao et al. (2023)
Proliferation, migration, invasion, and apoptosis	CircANO5	miR-21–5p/LIFR	lidocaine	Guan et al. (2022)
Apoptosis and ferroptosis	Circ0008035	miR-302a/E2F7	Dexmedetomidine	Gao and Wang (2023)
Proliferation and invasion	CircPVT1	miR-195–5p/ETS1	Propofol	Sui et al. (2020)
Survival, metastasis, apoptosis, and glycolysis processes	Circ0000376	miR-145–5p	bupivacaine	Ju et al. (2020)
Proliferation, migration, and invasion	CircPDIA4	MAPK	ERK inhibitors	Shen et al. (2023)

### 1.1 CircRNAs affect the invasion and migration of gastric cancer cells to regulate their sensitivity to drugs

The migration and invasion of cancer cells play a crucial role in the occurrence and development of various cancers, including gastric cancer. One of the primary factors contributing to the malignant progression and drug resistance in gastric cancer is the invasion and metastasis of tumor cells. Exosomes play a crucial role in the occurrence and development of gastric cancer as intercellular communication mediators. Circ0063526 could be transmitted to cisplatin sensitive gastric cancer cells through exosomes, thereby spreading cisplatin resistance. Knockdown circ0063526 in the exosomes prevented cisplatin resistance by inhibiting the migration, invasion, and autophagy of gastric cancer cells (Yang et al., 2022). CircLRCH3 promoted oxaliplatin resistance in gastric cancer cells by regulating the miR-383-5p/FGF7 axis, affecting cell proliferation, invasion, and apoptosis (Xiang et al., 2023). In the cancer tissues of patients with gastric cancer, circVAPA expression was elevated. The reduction of circVAPA reduced the ability of proliferation, migration, invasion of gastric cancer cells, which lessened their resistance to cisplatin (Deng et al., 2021). Compared with normal gastric tissue/cells, circ0026359 is highly expressed in gastric cancer tissue/cells, and its expression level is higher than that of cisplatin sensitive gastric cancer tissue/cells. Circ0026359 improved cisplatin resistance in gastric cancer cells by regulating cell invasion and migration through the miR-1200/POLD4 pathway (Zhang et al., 2020a). Circ0081143 was highly expressed in the tissues and cell lines of doxorubicin resistant gastric cancer. Circ0081143 regulated the migration, invasion, and proliferation of gastric cancer cells, thereby affecting the drug sensitivity of these cells to doxorubicin (Ou et al., 2022). Exosomal circ0032821 regulated the proliferation, migration and invasion of oxaliplatin sensitive gastric cancer cells through the miR-515-5p/SOX9 axis, enhancing oxaliplatin resistance in gastric cancer cells (Zhong et al., 2021). Knockdown of circLDLRAD3 regulated the invasion and proliferation of gastric cancer cells through miR-588/SOX5, reducing cisplatin resistance in gastric cancer cells (Liang et al., 2023). Zhou et al. (2021) revealed that downregulation of circPLEC inhibited the migration and invasion of gastric cancer cells, promoted apoptosis of paclitaxel resistant gastric cancer cells, and weakened their resistance to paclitaxel. The absence of circPDSS1 reduced the resistance of cisplatin resistant gastric cancer cells, mainly by affecting cell migration, invasion, and proliferation, and inducing cell apoptosis (Wang et al., 2023). The above research proves that migration and invasion play a crucial role in the occurrence and development of gastric cancer, and are also one of the main reasons for the malignant progression and drug resistance of gastric cancer. Multiple circRNAs are abnormally expressed in the occurrence and development of gastric cancer, regulating the invasion and migration process, and thus responding to the drug resistance of gastric cancer cells, which may become a new therapeutic target.

### 1.2 CircRNAs affect the proliferation of gastric cancer cells to regulate their sensitivity to drugs

Uncontrolled cell proliferation is one of the main characteristics of cancer. Uncontrolled proliferation of cancer cells is also one of the main mechanisms underlying cancer progression and drug resistance. Chen et al. (2021) found that circDLG1 was markedly upregulated in anti-PD-1-treated tissues and distant metastatic gastric cancer lesions. CircDLG1 acted as a miRNA sponge to increase CXCL12 expression and interact with miR-141-3p, which aided in the progression of the cancer and its resistance to anti-PD-1 based therapy (Chen et al., 2021). Circ004413 promoted cell proliferation of gastric cancer cells and resistance to 5-Fu through miR-145-5p (Zhou et al., 2022). The interaction between FOXM1 and circ-NOTCH1 suppressed the expression of circ-NOTCH1 to inhibit the proliferation and invasion of gastric cancer cells, and regulated the expression of resistance genes to affect the drug resistance of gastric cancer cells (Ge et al., 2023). CircAVCF increased in cisplatin resistant gastric cancer tissues and cells. CircAVCF regulated gastric cancer cell proliferation and metastasis through miR-1205/FGFR1, enhancing cisplatin resistance of gastric cancer cell (Zhang et al., 2021). Gastric cancer tissues and cells resistant to cisplatin had higher levels of Circ0012774. Inhibiting Circ0012774 increased the sensitivity of gastric cancer to cisplatin by influencing cell proliferation through the interaction of miR-637/CDX2 (Xu et al., 2022). In chemotherapy resistant tissues and cisplatin resistant gastric cancer cells, the expression of circPVT1 increased. Inhibition of circPVT1 enhanced the sensitivity of cells to cisplatin, inhibits the cell viability, proliferation, and induces apoptosis of cisplatin resistant gastric cancer cells (Wang et al., 2021). CircMTHFD2 affected gastric cancer cell resistance to pemetrexed by regulating miR-124 expression and cell survival rate (Xu et al., 2019). CircMCTP2 modulated various processes, including cell proliferation to affect the sensitivity of gastric cancer cells to cisplatin (Sun et al., 2020). It is reported that circASAP2 regulates gastric cancer cells proliferation, migration, invasion, and apoptosis through the miR-330-3p/NT5E axis, inhibiting gastric cancer cells sensitivity to cisplatin drugs (Sun et al., 2021). Circ0001546, as a miRNA-421 sponge, regulated cell proliferation through ATM/Chk2/p53 dependent pathways and inhibited resistance of gastric cancer cells to Oxaliplatin (Wu et al., 2020). The uncontrolled proliferation of cells is one of the basic characteristics of tumors such as gastric cancer, and it is also one of the main mechanisms of drug resistance in gastric cancer. Many research results have found that circRNA can regulate the proliferation ability of gastric cancer cells and affect their sensitivity to anti-tumor drugs. Subsequent research could focus on developing anti-tumor drugs targeting circRNAs that regulate gastric cancer cell proliferation, which may be a new research hotspot.

### 1.3 CircRNAs affect autophagy of gastric cancer cells to regulate their sensitivity to drugs

Autophagy is an evolutionarily conserved lysosomal dependent self degradation pathway that plays an important role not only in maintaining genomic integrity and maintaining internal environmental stability, but also closely related to cancer resistance.

In gastric cancer tissues and cells, CircCUL2 was markedly downregulated, and through miR-142-3p/ROCK2 mediated cell autophagy, it controlled the drug susceptibility to cisplatin (Peng et al., 2020). The expression of circCPM significantly increased in 5-FU resistant gastric cancer cell lines and tissues. Both *in vitro* and *in vivo* chemotherapeutic sensitivity was significantly increased by silent circCPM. By binding to miR-21-3p, CircCPM increased PRKAA2 expression, which in turn promoted autophagy and resistance to chemotherapy (Fang et al., 2022). Compared with cisplatin sensitive gastric cancer cells and tissues, circMCTP2 was downregulated in drug-resistant gastric cancer cells and tissues. CircMCTP2 regulated cell autophagy, apoptosis, and proliferation, affecting the sensitivity of gastric cancer cells to cisplatin (Sun et al., 2020). Chen et al. (2023) found that exosomal circ0091741 derived from gastric cancer cells regulates miR-330-3p/TRIM14/Dvl2/Wnt/β-catenin axis to induce autophagy and Oxaliplatin resistance in gastric cancer cells. The expression of PLAG1 and ATG12 was activated by CircPOFUT1-regulated miR-488-3p, which improved the malignant phenotype and autophagy-related chemical resistance in gastric cancer cells (Luo et al., 2023).

Autophagy plays multiple roles in cancers such as gastric cancer. On the one hand, protective autophagy can promote the survival of drug-resistant cells. On the other hand, autophagy as a programmed death mechanism can directly promote the death of drug-resistant tumor cells. Focusing on the research progress of autophagy related circRNAs in gastric cancer chemotherapy resistance can provide new ideas for overcoming tumor multidrug resistance.

### 1.4 CircRNAs affect apoptosis of gastric cancer cells to regulate their sensitivity to drugs

Due to various environmental and genetic factors, cells lose the ability to undergo apoptosis, which is one of the key factors in cancer occurrence and development. Stimulating and restoring the ability of cancer cells to undergo apoptosis is an effective way to prevent and treat tumors.

CircRNA plays a critical role in regulating the resistance of gastric cancer to cisplatin. CircUBAP2 promoted apoptosis and inhibits cisplatin resistance in gastric cancer cells through miR-300/KAT6B axis (Cheng et al., 2023). CircCCDC66 inhibited gastric cancer cell apoptosis by targeting miR-618 and BCL2, thereby reducing the sensitivity of gastric cancer cells to cisplatin. The development of cisplatin resistance was regulated by CircCDC66, which holds potential as a therapeutic target for gastric cancer (Zhang et al., 2020b). It was reported that circAVCF levels increase in gastric cancer tissues and cells. Silencing circAVCF caused gastric cancer cells to undergo apoptosis and prevents them from becoming resistant to cisplatin. Mechanistically, circAVCF enhanced gastric cancer resistance to cisplatin through miR-1205/FGFR1 (Zhang et al., 2021). Lv et al. (2020) found that the expression of cir0000520 was reduced in gastric cancer tissues and cells, regulating gastric cancer cells apoptosis through the PI3K/Akt pathway, thereby affecting gastric cancer cell sensitivity to herceptin. Circ0012774 was highly expressed in gastric cancer tissues and cells, and affected cisplatin sensitivity by regulating cell apoptosis through miR-637/CDX2 (Xu et al., 2022). Knockdown circ0063526 in the exosomes inhibited cisplatin resistance by inhibiting the migration, invasion, and autophagy of gastric cancer cells (Yang et al., 2022). The expression of circPVT1 increased in gastric cancer cells. The inhibition of circPVT1 increased the sensitivity of gastric cancer cells to cisplatin, inhibited the proliferation of cisplatin resistant gastric cancer cells, and induced gastric cancer cells apoptosis (Wang et al., 2021). CircLRCH3 promoted oxaliplatin resistance in gastric cancer cells by affecting cell apoptosis, proliferation and invasion (Xiang et al., 2023). By increasing gastric cancer cells’ apoptosis, the decrease of circVAPA lessened gastric cancer cells’ resistance to cisplatin (Deng et al., 2021). CircCEA protected gastric cancer cells from stress-induced apoptosis by acting as a protein scaffold and interacting with p53 and CDK1 proteins. Targeted circCEA combined with doxorubicin caused more cell apoptosis, reduced tumor volume, and alleviated the side effects of doxorubicin (Yuan et al., 2022). By negatively targeting miR-30a-5p, CircPVT1 knockdown induced apoptosis and decreased autophagy or invasion in gastric cancer cells, consequently impeding cisplatin resistance in gastric cancer cells (Yao et al., 2021). By regulating ZEB1 expression through miR-124-3p, CircPVT1 affected apoptosis and invasion of gastric cancer cells to promote their resistance to paclitaxel (Liu et al., 2019). Through miR-543/PHLPP2, circ0001017 regulated apoptosis and migration of gastric cancer cells, making cisplatin resistant gastric cancer cells sensitive to chemotherapy (Zhang et al., 2022a). Knockdown of circASAP2 promoted sensitivity to cisplatin, cell apoptosis, and inhibited the proliferation, migration, and invasion of cisplatin resistant gastric cancer cells *in vitro*, while inhibiting tumor growth *in vivo* (Sun et al., 2021). Apoptosis escape (increased cell anti-apoptotic ability) is one of the important mechanisms for the decreased sensitivity of cancer drugs in tumors such as gastric cancer. CircRNA plays a critical role in regulating the resistance of gastric cancer. Focusing on the role of apoptosis related circRNAs in gastric cancer resistance can reveal new molecular mechanisms and provide new strategies for the treatment of gastric cancer resistance or enhancing anti-cancer drug sensitivity.

### 1.5 CircRNAs affected other physiological and pathological processes to regulate drug resistance in gastric cancer cells

DNA damage repair refers to the phenomenon of restoring the structure of DNA molecules within cells after being damaged by various enzymes. Research on DNA damage repair helps to understand the mechanisms of cancer occurrence and development, as well as the reasons for cancer cell resistance. Intracellular cisplatin mainly binds to nuclear DNA and can interact with mitochondrial DNA. In gastric cancer cells, mitochondrial DNA has the ability to regulate cell apoptosis. The data from Huang et al. indicate that circAKT3 enhances the resistance of gastric cancer cells to cisplatin by promoting DNA damage repair and inhibiting apoptosis through miR-198/PIK3R1 (Huang et al., 2019).

Cancer stem cells refer to a small portion of cancer cells that can maintain self-renewal and differentiation abilities, and are considered the origin and driving cells of cancer, including gastric cancer. It has been established that cancer stem cells are the primary cause of cancer metastasis, recurrence, and treatment failure. They also play a critical role in chemotherapy resistance and the malignant progression of cancer. CircFAM73A enhanced cisplatin resistance by promoting gastric cancer stem cell properties through the miR-490-3p/HMGA2 and HNRNPK/β-catenin stabilization (Xia et al., 2021). Circ50547 acted as a sponge for miR-217 to regulate the expression of HNF1B, affecting the cancer stem cell-like properties, proliferation, migration, and invasion of gastric cancer cells, ultimately enhancing the drug resistance of gastric cancer cells (Zang et al., 2024).

Increasing evidence has identified the important role of macrophage polarization in chemoresistance. Qu et al. (2023) revealed that CircSOD2 polarized macrophages towards the M1 phenotype by targeting the miR-1296/STAT1 axis, thereby reducing cisplatin resistance in cancer cells.

The term “immune escape mechanism” describes the ways in which cancer cells use different strategies to avoid being identified and eliminated by the host immune system. This is a major factor in the initiation, progression, and spread of cancer. The immune escape mechanism of cancers is an important obstacle to cancer treatment and a significant reason for drug resistance in tumor immunotherapy. Research suggested that cir136666 drives PD-L1 phosphorylation through the miR-375/PRKDC signaling axis, promoting immune escape and affecting the therapeutic effect of immune preparations (Miao et al., 2023).

DNA damage repair, changes in cancer stem cells activities, immune escape, macrophage polarization, etc., all play a role in the drug resistance of gastric cancer cells. Revealing the role and mechanism of circRNA in these processes can provide a new approach to enhance the sensitivity of gastric cancer cells to anti-tumor drugs.

### 1.6 CircRNAs affect the efficacy of other drugs in the treatment of gastric cancer

Numerous phytochemicals and anesthetics, in addition to first-line chemotherapy medications, are crucial in the prevention and treatment of gastric cancer. Studies have found that circRNA regulates the sensitivity of these drugs in gastric cancer cells.

Through the miR-145-5p/PBX3 axis, circTRPM7 improved the inhibitory effect of sinomenine on the development and metastasis of gastric cancer cells (Yan et al., 2023). Icariin inhibited gastric cancer cell growth by regulating the cir0003159/miR-223-3p/NLRP3 signaling axis (Zhang et al., 2022b). CircSFMBT2 regulated the effects of paeonol on gastric cancer cell proliferation, apoptosis, migration, invasion, and glutamine through miR-665 (Li et al., 2020). CircCDR1 and circHECTD1 via miR-7-5p/REGγ and miR-137/PBX3 axis affected the role of Diosbulbin-B in the prevention and treatment of gastric cancer (Li et al., 2019; Lu et al., 2021). CircDLST/miR-489-3p/EIF4A1 regulated the proliferation and apoptosis of gastric cancer cells, affecting the anti-gastric cancer effect of Astragaloside IV (Li et al., 2022). Non coding RNAs such as circRNA ITGA3 regulated the invasion and migration of gastric cancer, and their efficacy in the prevention and treatment of gastric cancer in matrine (Gao et al., 2023). CircANO5/miR-21-5p/LIFR promoted the anti-gastric cancer effect of lidocaine by regulating the proliferation, migration, invasion, and apoptosis of gastric cancer cells (Guan et al., 2022). Circ0008035/miR-302a/E2F7 regulated apoptosis and ferroptosis in gastric cancer cells, which enhanced the inhibitory effect of dexmedetomidine on the occurrence and development of gastric cancer (Gao and Wang, 2023). Propofol inhibited the proliferation and invasion of gastric cancer cells by regulating the circPVT1/miR-195-5p/ETS1 axis, but enhanced the apoptosis of gastric cancer cells (Sui et al., 2020). Circ0000376/miR-145-5p played a crucial regulatory role in the inhibition of cancer cell survival, metastasis, and glycolysis processes, promoting cell apoptosis by bupivacaine (Ju et al., 2020). CircPDIA4 was significantly upregulated in malignant gastric cancer tissues and is associated with low survival rates in gastric cancer patients. The high expression of circPDIA4 promoted distant metastasis in various mouse xenograft models and accelerated invasion *in vitro*. It is worth noting that circPDIA4 deficiency enhanced the sensitivity of gastric cancer cells to ERK inhibitors, affecting the treatment of gastric cancer (Shen et al., 2023).

Cancers, including gastric cancer, are one of the leading causes of death worldwide. More and more phytochemicals and small molecule drugs are showing good effects in the tumorigenic process of gastric cancer and other tumors. We need to constantly discover new phytochemicals and small molecule drugs to enhance the therapeutic effect of gastric cancer and reduce side effects.

## 2 Conclusion and future perspective

Gastric cancer does not have obvious and specific early symptoms, and there is also a lack of specific and effective early diagnostic markers in clinical practice. Since gastric cancer is frequently diagnosed at the advanced stage, there has been much discussion over how sensitive this disease is to treatment medications. More and more circRNAs have been identified to be associated with drug resistance in gastric cancer. As shown in Figure 1, the drug resistance related circRNAs in gastric cancer are summarized. Targeting these abnormally expressed circRNAs is a promising method for reversing drug resistance in gastric cancer. Reversing drug resistance in gastric cancer has been studied through overexpression of gastric cancer suppressive circRNA or knockdown of oncogenic circRNA using lentivirus, small interfering RNA, short hairpin RNA, gene editing techniques, and so on. To overcome drug resistance in advanced gastric cancer, circRNA-based therapeutic interventions in combination with targeted therapy, immunotherapy, or conventional chemotherapy may be a promising approach. However, the exact molecular mechanism of circRNA in gastric cancer resistance has not yet been fully revealed, and the safety of treatment methods based on circRNA also needs to be comprehensively evaluated. On the other hand, numerous circRNAs have been found to be associated with drug resistance and insufficient sensitivity to therapeutic drugs in gastric cancer. Accurately selecting key target circRNAs from a large number of candidates circRNAs remains a challenge. Additionally, research and validation on population tissue samples must be carried out prior to clinical use. Improvements must be made to the techniques for identifying and utilizing circRNAs associated with drug resistance in gastric cancer. Specific aspects such as repeatability, specificity, and sensitivity require additional assessment. Furthermore, exosomes have the ability to transport active components that contribute to both the onset and progression of gastric cancer as well as intercellular communication. It is necessary to clarify whether circRNAs related to gastric cancer resistance can affect the microenvironment of gastric cancer through extracellular vesicles and play a key role in gastric cancer resistance. At the same time, it is possible to solve gastric cancer resistance by intervening in the extracellular vesicle pathway.
